# Reasons for admission and post-release survival of UK rehabilitated herring gulls (*Larus argentatus*) from 1999 to 2024

**DOI:** 10.1017/awf.2025.10047

**Published:** 2025-10-22

**Authors:** Richard Thompson, B. Louise Chilvers, Martyn J. Stenning

**Affiliations:** 1 Royal Society for the Prevention of Cruelty to Animals, Wildlife Department, Horsham, West Sussex RH13 9RS, UK; 2 Wildbase, School of Veterinary Sciences, Massey University, Palmerston North, New Zealand; 3 (Associate) School of Life Sciences, University of Sussex, Brighton BN1 9QG, UK

**Keywords:** Admission criteria, animal welfare, herring gulls, HPAI, rehabilitation, release, survival

## Abstract

Globally, millions of animals transition through wildlife rehabilitation facilities annually. Data recorded at these facilities can be used to quantitatively assess factors which result in the animals’ admittance, treatment, release, and survival, and how impacts such as high pathogen avian influenza (HPAI) has altered these parameters. Twenty-five years of records of herring gull (*Larus argentatus*) admittances into RSPCA Mallydams Wood Wildlife Rehabilitation Centre, Hastings, UK (between 1999 and 2024) were reviewed to determine admission factors and their impacts on the number of days in care and the likelihood of release. Additionally, for the years 1999 to 2010, data were collected on days of post-release survival and distances from the centre travelled from ringed and released birds. During that 25-year period, 17,334 herring gulls were admitted into the Mallydams Centre with 9,013 released, and 2,796 ringed and released between 1999 and 2010. Release rates varied significantly with the category of problem identified at admission. Wild nesting herring gulls, even without the impact of HPAI, have been declining throughout the UK, and the additional anthropogenic pressures on urban gull populations have resulted in a documented national decline in the species. Rehabilitating and returning birds to the wild has shown to be important both for their animal welfare and population, as well as helping identify the impact of HPAI on local urban populations of all relevant species. Results from this research can be utilised to adapt training and resources at rehabilitation centres and determine euthanasia protocols to optimise animal welfare along with release and survival success.

## Introduction

Wildlife rehabilitation is defined as “*the treatment and temporary care of injured, diseased, and displaced animals, and the subsequent release of healthy animals to appropriate habitats in the wild*” (Miller 2012). There is continual debate regarding the extent to which wildlife rehabilitation should occur and the identification of both individual animals and species that can, or should, be treated, which will survive treatment and be released back to the wild. However, at the same time as this debate occurs, public demand and expectation, throughout the world, for wildlife rehabilitation to occur has increased (Karesh 1995; Miller *et al.* 2023). In England and Wales, the estimated annual admission of traumatised or orphaned wild animals into rescue centres is in the order of 270,000 and 300,000 individuals, with the Royal Society for the Prevention of Cruelty to Animals (RSPCA) alone rescuing or collecting 170,000 wildlife casualties per year prior to significant Avian Influenza outbreaks across Europe from 2021 (Verhagen *et al.* 2021). Grogan and Kelly (2013) stated: “*The RSPCA believes that the welfare of wildlife casualties can be improved by investigating which injuries or illnesses are most likely to result in a successful release for each species, and by collecting data on post-release survivorship*”. This research analyses 25 years of records of herring gull (*Larus argentatus*) admittance into the RSPCA Mallydams Wood Wildlife Rehabilitation Centre, Hastings, UK to: (1) determine what impact admission factors have on the likelihood of release; (2) investigate days of post-release survival for this species between 1999 and 2010; and (3) compare admission data for 1999 to 2022, with the years of highest impact and greatest restrictions on the RSPCA ability to admit wild birds because of High Pathagen Avian Influenza (HPAI), notably 2022 and 2023.

Intervention has been deemed the correct course of action when presented with a sick or injured wild animal within the UK, however, the question of whether intervention is always necessary and appropriate remains. The action of capture and confinement has a negative effect on wild animals, and poor capture technique and an unsuitable transport box could cause morbidity or mortality (Dmytryk 2012). There are isolated incidences where wild animals adjust to injuries or illness and survive without human intervention. A study of *L. fuscus* gulls in The Netherlands (Camphuysen 2011) after a mystery oil spill affected birds during the breeding season, showed that small patches of oil will disappear through ‘weathering off’ or deliberate removal from routine preening by the bird. In 46 cases of colour-ringed birds observed, only two died, but the majority continued to incubate eggs and rear chicks. There are observations of wild gulls and waders surviving in the wild having a missing leg or the lower part of the tarsus removed (R Thompson, personal observation 2014), most probably due to fishing line ligation or as fledglings in the nest. These birds will adapt to the injury by spending time on water or flying, but over time an injured limb may atrophy and fall off.

Herring gulls are one of the species most commonly admitted into rehabilitation centres in Great Britain (12% of admittance in a 10-year period; Mullineaux & Pawson 2024). Undeniably, there are conflicting attitudes to gulls around the world, increasingly from people who live in cities and towns where urban nesting colonies exist. The increase of gulls in urban habitats (Rock 2005; Pais de Faria *et al.* 2021) has led them to be categorised by some as a nuisance or pest species. Most gull species, including the herring gull, are typically found at sea level and are associated with marine environments, but more recently, gulls are found inland, frequently seen in urban areas and on landfill sites over 85 km from the coast (Rock 2005; Pais de Faria *et al.* 2021). Gulls are adaptable and opportunistic in diet preferences, taking carrion, invertebrates (marine and terrestrial), fish, and young and adult birds of other species (Cramp & Simmons 1983). Herring gulls have adapted to feeding on anthropogenic waste at landfill sites, fishing fleet discards, unwanted foodstuffs in coastal towns, sewage outlets, livestock food storage areas, and from being fed by humans (Huppop & Wurm 2000; Garthe & Scherp 2003; Rock 2005; Pais de Faria *et al.* 2021). Herring gull preferred breeding habitats are varied; coastal cliffs and stacks, rocky and grassy islands, sandy beaches, gravel bars, salt marshes and limestone outcrops (del Hoyo *et al.* 1996). However, it is being increasingly noted that they are also nesting on roof tops in urban areas (Pais de Faria *et al.* 2021; Burnell *et al.* 2023). The International Union for the Conservation of Nature (IUCN) list herring gulls as a species of least concern but decreasing (BirdLife International 2021). The UK Bird of Conservation Concern lists herring gulls as red-listed (the highest threat classification) due to a > 50% decline in the coastal breeding population (Stanbury *et al.* 2021). The global breeding population is estimated to number 531,000 to 608,000 pairs, which equates to approximately 1,060,000 to 1,220,000 mature individuals and 1,590,000 to 1,830,000 total individuals (BirdLife International 2021). The UK population estimate is 130,000 pairs (Stanbury *et al.* 2021). The decline appears to be partly linked to improved waste management practices and reduced fishery discards, indicating the species population may be readjusting to a lower level similar to when it was unable to take advantage of anthropogenic and unsustainable food sources (Wilhelm *et al.* 2016). The decline in population is most likely attributable to low recruitment of juveniles into the population, as juveniles are especially reliant on waste and fishery discards and are therefore not surviving to enter the population (Wilhelm *et al.* 2016). Reduction in discards has impacted greatly on food availability throughout parts of the species’ range that had seen previously rapid increases in the last century. Further, trophic level shifts have impacted fishing activity and catch size, which appears to be directly related to colony reproductive success: lower catches are resulting in lower breeding success and the decline of colonies in the UK (Foster *et al.* 2017; Stanbury *et al.* 2021). Changes to land-fill management across northern Europe may be detrimental to winter survival of herring gulls (Olsson & Hentati-Sundberg 2017; Shlepr *et al.* 2021). The species is also vulnerable to collisions with wind turbines (Bradbury *et al.* 2014) with 39 individuals out of 114 recorded dead found to have collided with such structures in an 11-year study covering 4.7 km of English coastline (Newton & Little 2009). Yet another threat to species numbers comes in the form of coastal oil pollution (Gorski *et al.* 1977).

Gulls are also shown to be highly susceptible to avian influenza (Melville & Shortridge 2006), with an estimated 7% decline in coastal breeding populations attributed to HPAI (Lean *et al.* 2023). Botulism reports have been on the increase in Britain over the last 45 years, and despite numbers of deaths lacking quantification, it is suggested as being a factor in the population declines in Britain (Coulson 2015). Herring gulls are vulnerable to being caught as bycatch in fisheries, including longlines, trawls and gillnets (Anderson *et al.* 2011; Zydelis *et al.* 2013). The species is protected under the African Eurasian Waterbird Agreement. All gull species and their eggs and nests are protected under the Wildlife and Countryside Act, UK, and are listed as a priority in the UK Biodiversity Action Plan.

This research examines 25 years of records of herring gull admissions at the RSPCA Mallydams Wood Wildlife Rehabilitation Centre, to investigate how reasons for admission and the centre’s triage, treatment and release protocols, impact the likelihood of herring gulls’ release, and for the period between 1999 to 2010 their days of post-release survival. These baseline data are then used to compare admission data with those years of high HPAI infections (2022 and 2023) and the changes in admissions and outcomes that occurred.

## Materials and methods

Records of herring gull admissions were studied for 25 years from the RSPCA Mallydams Woods Centre, East Sussex, UK. For all gulls brought into the centre between the start of 1999 and June 2022, admissions were treated using the national protocol for the rehabilitation of all members of the family *Laridae*, written by RSPCA staff (see Supplementary material). In summary, the protocol included all wildlife casualties that were accepted through the reception area where a record for that individual was started in a Microsoft® Access database. Mandatory fields on the database were species, date, age, location found, county, reason for admission and finder’s name and address. The age of gulls on admission was based upon assessments by wildlife staff and volunteers. In the infrequent cases of aberrant plumage (unusual and non-typical feather colouration or feather growth) or birds in moult, specifically during autumn/winter months (August–November), identification guides and reference books were utilised (Baker 1993; Olsen & Larsson 2004; Svensson *et al.* 2009). Age categories (using the British Trust for Ornithology [BTO] age codes for release) included: Nestling, Fledgling, Juvenile, Immature, Adult and Not known. All birds were reassessed on release to confirm age classes (also removing the ‘Not known’ classification) at ringing. On admission, each animal was allocated a unique case number, and all treatment, observations and final outcomes of admission were written up into the database under that case number which is engraved onto and fitted to the bird via a temporary aluminium leg ring. Birds with obvious severe injuries, for example, compound leg or wing fractures, were euthanased upon admission to alleviate further suffering. All other animals were administered first aid and taken through to housing within the centre. Subsequently, all birds not orphans or inexperienced juveniles; juvenile birds found fully fledged but unable to sustain continuous flight or in a hazardous environment, were re-examined by a veterinarian to diagnose any trauma or disease via radiography, blood tests and anaesthesia. The reasons for admission were immediately recorded and a treatment care plan established. Reasons for admission would include: attacked by another gull; attacked by other animal; inexperienced juvenile; orphan; disease/weakness; fishing litter; caught/entangled; shot; poison/botulism; poison/neurological; oiling/other contaminant; injury/collision; and legal case animal (birds admitted under this category may form part of a prosecution case and could be from a range of causes such as shooting, trapping, poisoning or failing to provide appropriate care or treatment).

In the period between June 2022 and December 2023, all admitted herring gulls were euthanased due to the HPAI epidemic, however, administration records of wildlife record number, species, date, age, admission reason, outcome, outcome date and days in care were still recorded. From 1 Jan 2024 protocols returned to the standard protocol listed above.

No specific licences were required for this work as it was a retrospective analysis of data recorded by the RSPCA as a part of their normal protocol.

### Ringing and re-sighting: 1999 to 2010

Permits were issued to Mallydams to ring birds from the BTO (Thompson 2013). All rehabilitated gulls released between 1999 and 2010 were fitted with a uniquely coded ‘nickel-chromium alloy’ ring size ‘G’ with an internal diameter of 11 mm to their right tarsus (Redfern & Clark 2001). In addition, a colour-ringing scheme (Darvic rings) was introduced in July 2000 using colours and codes varying year-to-year. The scheme was allocated by Peter Rock, the large white-headed gull coordinator, and registered with EURING so that colour sightings could be reported directly to this project. All unique colour rings also had individual metal rings. This enabled remote re-sighting records of birds to be linked back to their Incoloy Ring.

Birds found dead or recovered alive and reported to the BTO generated a ringing recovery report. These results were added to the individual bird’s RSPCA database case file. Additional observations came from the European colour-ringing birding website http://www.cr-birding.org/. Information from each recovery or sighting included ring number, Darvic code, recovery code, date seen, place seen, longitude and latitude and observer was also added to the individual bird’s RSPCA database case file. Only data that had both a date and location were included in the records.

For calculating the temporal and spatial intervals from observations between release date and re-sighting, the BTO recovery form automatically calculates the survival days, distance and compass direction travelled from the ringing site to the place of recovery. For re-sightings and recoveries reported directly to the ringer, the distance travelled and travel direction were calculated manually for the bird. Where the place of recovery or sighting is known, but not the coordinates, the place name is entered into the search facility on the Google Earth© website to retrieve the correct coordinates in time format (degrees, minutes and seconds). Using Excel® trigonometry, the distance travelled was determined from a great circle calculator using non-Euclidean geometry (Consulting 2011). The formula was tested on known coordinates and distances from BTO recovery forms, which provided comparable accuracy (Thompson 2013).

### Analysis

IBM SPSS Statistics version 19 were used to undertake Chi-squared tests with pairwise comparisons using Bonferroni correction were used to analyse the proportion of birds released for each of the main admission categories between 1999 and 2024, and *t*-tests to compare mean survival days and mean distance travelled for adult versus non-adult birds between 1999 and 2010.

## Results

### Annual trends in gull admissions

**Figure 1.**
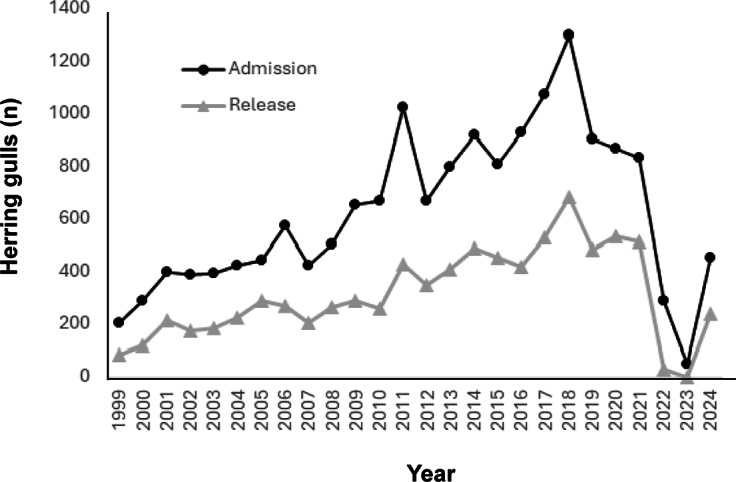
Annual numbers of herring gulls (*Larus argentatus*) admitted and released from RSPCA Mallydams Wood Wildlife Rehabilitation Centre, Hastings, UK, between 1999 and 2024.

In the 25 years (1999–2024), a total of 17,334 herring gull admissions were recorded, with admissions increasing most years up to 2018, dropping and stabilising 2019 to 2021 and then dropping significantly during the HPAI outbreak (2022 to 2023) and increasing again in 2024 when HPAI restrictions were lessened (Figure 1).

**Figure 2.**
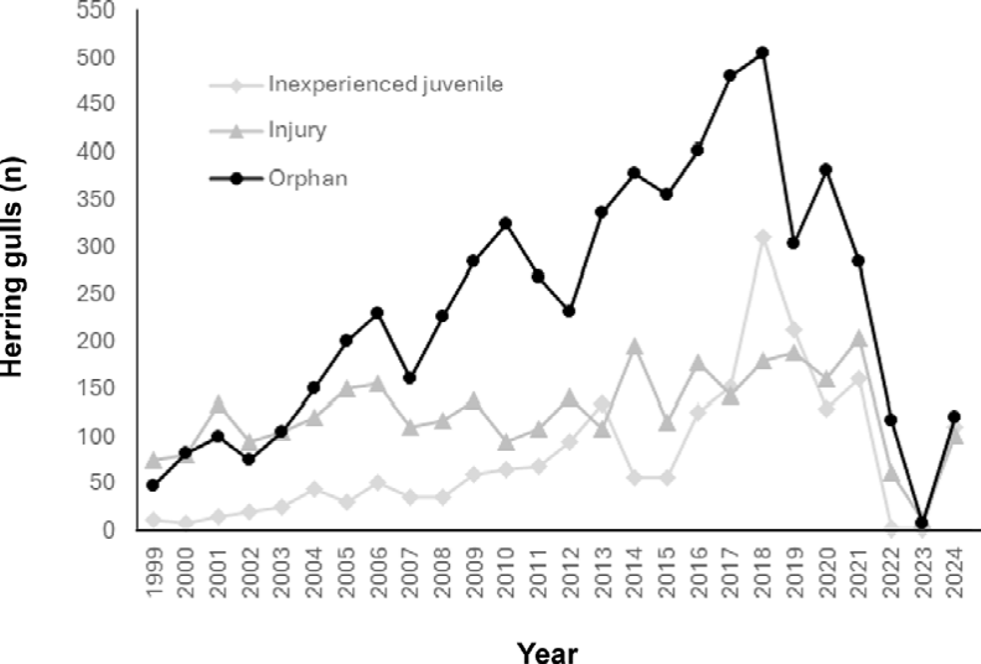
Annual numbers of herring gulls (*Larus argentatus*) admitted to the RSPCA Mallydams Wood Wildlife Rehabilitation Centre, Hastings, UK, in the common categories: Orphan; Injury; and Inexperienced juveniles between 1999 and 2024.

**Figure 3.**
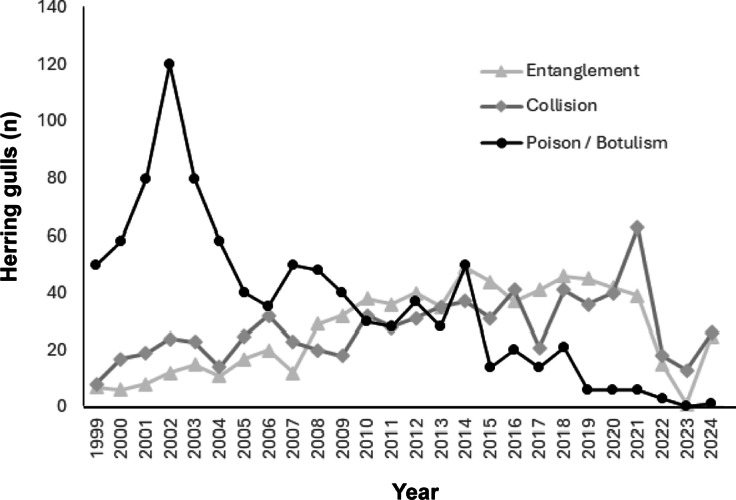
Annual numbers of herring gulls (*Larus argentatus*) admitted to the RSPCA Mallydams Wood Wildlife Rehabilitation Centre, Hastings, UK, in the less common categories: Poison/Botulism, Collision and Entangled, between 1999 and 2024.

There were eleven main categories within three distinct groups for reasons of admissions. Group 1 (common > 10% of admissions), includes Orphan, Injury, and Inexperienced juvenile (Figure 2). Group 2 (less common between 2 and 10% of admissions), included Poison/Botulism, Collision, and Caught/Entangled, and group 3 (rare 1 to 2% of total admissions; Table 1; Figure 3). All other problem categories were less than 1%. Combined, these three groups’ percentage of total admissions were 94.1%: common 71.7%; less common 16.4%; and rare 6% (Table 2).

**Table 1.** The total admittance, numbers that died or were euthanased, numbers released and release rate of herring gulls (*Larus argentatus*) admitted to the RSPCA Mallydams Wood Wildlife Rehabilitation Centre, Hastings, UK, between 1999 and 2024. *Post-hoc* Chi-squared analysis showing release rates for problem categories in descending order with five homologous groups identified by lowercase letters is shown in the far-right column

Categories	Total admitted	Died or Euthanased	Released	Release rate	Post hoc
Inexperienced	1,967	497	1,470	74.8	a
Orphan	5,933	1,824	4,109	69.3	a
Entangled	671	264	407	60.7	e
Botulism	903	462	441	48.9	e
Other attack	176	94	80	45.5	e
Neurological	274	157	117	42.8	e
Weakness	191	131	50	26.3	d
Collision	695	531	164	23.6	b
Injury	3,186	5,120	626	19.7	b
Shot	157	131	26	16.3	c

**Table 2.** The frequency, percentage and group definition for all problem categories for herring gulls (*Larus argentatus*) admitted to the RSPCA Mallydams Wood Wildlife Rehabilitation Centre, Hastings, UK, between 1999 and 2024. Admissions arranged in descending order

Category	Frequency	% of total	Occurrence
Orphan	5,933	38.3	Common
Injury	3,189	21.8	Common
Inexperienced juvenile	1,967	11.6	Common
Poison/Botulism	903	7.5	Less Common
Collision	695	4.6	Less common
Caught/entangled	671	4.3	Less common
Poison/ Neurological	274	1.6	Rare
Fishing litter	152	1.1	Rare
Shot	157	1.1	Rare
Attacked by other animals	176	1.1	Rare
Weakness	191	1.1	Rare

During 2023, when admissions were highly restricted due to HPAI, of the 50 admissions only three categories recorded more than one admission, those being: 26% collision, 22% injury and 20% other.

### Seasonal and age differences in admission categories

Each of the problem categories showed seasonal changes in the peak months of admission. The increase in admissions for all categories corresponds to the intake of nestling and inexperienced juvenile birds from May to August and adults suffering injury or misadventure during the breeding season. Admissions of predominantly adults suffering from botulism, were normally from May to September, with peaks in July and August. Peaks in adult admissions correspond to pre-breeding, breeding and post-breeding activity. Categories with the greatest admissions during the months June to August were ‘collision’ and ‘caught and entangled’. Noticeable increases in shot birds occurred during pre-breeding courtship in May, and those in August include ‘fishing litter’.

There was variation in admitted birds relative to age throughout the 25 years (Figures 2 and 3). The number of birds admitted under the categories: juvenile, fledgling, and nestling showed the greatest variation (Figure 2). Little variation was seen in the admission of adult birds throughout the study, apart from instances where significant outbreaks of botulism were close by (Figure 3). Seasonal variation in age admissions shows a predictable increase in numbers during the breeding months of May to August. Successive age groups: nestling, fledgling and juvenile correspond to the maturation of chicks which progress through the summer months. In July, the highest numbers of juvenile birds were admitted when young birds were beginning to fledge.

### Factors affecting release and survival

The number of days in care ranged from 1 to 376 days for all admissions, with a mean of 19.5 days. For those birds not released, the average was 8.3 days, which includes birds euthanased on admission or within 48 h; these included all admissions in 2023 and those for the second half of 2022. Birds that were released had a mean time in care of 28 days.

The number of birds recorded released was n = 9,013 or, on average, 52%, with a yearly range varying from 0% for the second half of 2022 and all of 2023 due to HPAI, to 59.6%. When the numbers for dead on arrival, humanely euthanased on admission or within 48 h are excluded, the combined years sample mean for released birds increased to 88%.

For that 88%, the percentage of released birds for each reason of admission category showed variation in survival to release rates. There was a significant difference in the relative proportion of birds released in the main problem categories (Chi-squared test: χ^2^ = 641; *P* < 0.0001). *Post hoc* analysis shows that five groups were identified, showing a significant difference in the rates of release (Table 1).

### Re-sightings and recoveries of rehabilitated and ringed birds from 1999–2010

A total of 2,796 gulls of all ages were ringed and released during the period from 1999–2010. The largest number of a specific age group ringed was 2,158 nestling, fledgling and juvenile gulls (75.6%), followed by 514 adults (18%). In total, 2,350 re-sighted birds were reported for these rehabilitated individuals, 1,472 (62%) were duplicate cases (two or more sightings of the same individual), and 878 (38%) individual recoveries. The re-sighting of the Darvic ringed birds in this study have come primarily from birdwatchers and private observers. The mean survival days and distances travelled for each age group are shown in Table 3. Overall, juvenile birds travelled the furthest with a mean distance 69.51 km and the highest mean survival days, 954.74 days, was seen in 1st year birds.

**Table 3.** Mean (± SD) distance travelled and survival days to the last event for all age groups of rehabilitated herring gulls (*Larus argentatus*) ringed and released from the RSPCA Mallydams Wood Wildlife Rehabilitation Centre, Hastings, UK, between 1999 and 2010

Age	Category	N	Minimum	Maximum	Mean	(± SD)
Juvenile	Survival days	691	2	4,197	719	706.0
	Distance/km	684	<1	1,454	70	106.9
1^st^ Year	Survival days	19	111	2,321	955	689.6
	Distance/km	19	2	166	44	44.8
2^nd^ Year	Survival days	17	7	1,443	660	476.5
	Distance/km	17	2	122	57	34.0
3^rd^ Year	Survival days	2	4	246	130	178.1
	Distance/km	1	19	19	19	–
Adult	Survival days	149	5	3,669	849	805.7
	Distance/km	149	2	1,873	59	159.9

Rehabilitated birds were separated into adult (n = 149) and non-adult (n = 728) groups for analysis. The mean (± SD) survival days for rehabilitated adults (848.77 [± 66] days) did not differ significantly (*t* = 1.950, df = 876; *P* = 0.051) from that for non-adult rehabilitated gulls (722.49 [± 26] days). Similarly, the distance travelled by the adult (58.69 [± 13.10] km) and non-adult group (68.46 [± 3.89] km) was not significantly different (*t* = –0.937, df = 876; *P* = 0.349).

For the 1,173 re-sightings where the distance was recorded, distances ranged from < 1 km to 1,454 km. The mean overall distance travelled was 74.3 (± 4.85) km. Re-sighted gulls were recorded over ten countries, the most numerous reports being from England (n = 739; 79.8%), followed by France (n = 154). The furthest distance recorded for an adult was 1,874 km, re-sighted at the Tampere Landfill site, near Taraste in Finland (61°30N 23°45E). For a juvenile the furthest sighting was a distance of 1,454 km near Palanga in Lithuania (55°55N 21°03E).

## Discussion

This research examined 25 years of records of herring gull admissions at the RSPCA Mallydams Wood Wildlife Rehabilitation Centre, UK. Results showed an increasing trend of admittance of herring gulls into the centre between 1999 and 2018, followed by a slight decrease, which corresponds with declines seen in the species (BirdLife International 2021). Records then show a significant decline in admission during the time (between June 2022 and December 2023) that HPAI restrictions within the UK constrained the RSPCA’s ability to accept any wild birds into their facilities. Overall, 52% of all admitted gull casualties were successfully returned back to the wild, a percentage slightly higher than recorded for wildlife across many rehabilitation centres in the UK (Mullineaux & Pawson 2024). When individuals euthanased within 48 h of admission (including 2022 and 2023 years) were removed, released birds increased to 88%. Annual fluctuations in mortality rates are related to: (1) increases in admissions owing to a sudden influx of nestling or fledgling birds due to unsettled or hot weather; (2) outbreaks of botulism in adult birds during the breeding season; and (3) HPAI restrictions (Figure 1).

Although an influx of nestlings and fledglings is an annual occurrence, numbers taken in vary year-on-year due to factors related to weather. However, understanding the impact time of year has on intake, and thus release rates for nestlings and fledglings, enables contingencies to be planned for in advance, i.e. by having extra pools and space during nesting/fledgling season. The fact that botulism outbreaks are not regular makes planning for them more of a challenge, however, greater understanding of the negative impact and known reduced survival rate of adults impacted by botulism, could lead to implementation of stricter triage protocols, for example, checking for additional secondary conditions in afflicted birds. HPAI is now resident within wild bird populations globally (except New Zealand and Australia), and as of April 2025 restrictions on bird movements and recommendations to the public not to handle wildlife of concern have increased again within the UK. These outbreaks are likely to continue and reduce the ability of rehabilitation centres to admit and care for wildlife. The impact of this may start to be seen in local populations with less survival and recruitment of young into the breeding population, as well as, increased animal welfare concerns. As seen from the 2023 data, herring gulls still incur injuries and collide with human infrastructure and vehicles regardless of HPAI outbreaks, leaving 100s of birds (Figure 2) injured and not being helped.

From this research, the mean release rate of herring gulls resembles published studies related to other rehabilitated birds in the UK throughout the last ten years (Mullineaux & Pawson 2024). Herring gulls have diverse reasons for admission relative to other species due to their anthropogenic foraging behaviours. This study showed that various admission categories in herring gulls offered different release rates, each related to the severity of injury or poor prognosis for release. The most successful release rates (74.8 and 69.3%) were for inexperienced juveniles and orphans, respectively which, combined, made up almost 50% of all admissions. These success rates tend to be due to these birds being fundamentally healthy, merely displaced with typically minor or no other injuries to speak of. Juvenile birds are usually quickly rehabilitated and released within 14 to 30 days. Orphan chicks, which are naïve, small, dependent birds, require more extended periods in care. However, chicks self-feed rapidly, and when kept with conspecific animals are less likely to imprint on humans and have high release and survival/re-sighting rates.

Reasons for admission related to anthropogenic causes, i.e. entanglement, show more variability in release rates. For ‘caught and entanglement’ which affects all age groups, release rates are high if injuries do not involve ligations around limbs (60.7%) with numbers of days in care ranging from 14 to 30. The lowest release rates are for birds that have been shot (16.3%). Adult birds were the group least likely to be released, since this age group suffered the greatest regarding encounters with multiple risks and hazards in the urban environment. Injuries sustained in this context are often life-threatening, with the most humane outcome being euthanasia at or soon after admission. This conclusion is in accordance with previous studies (Grogan & Kelly 2013) that found animals with complex injuries such as fractures or deep tissue wounds have low release rates and tend to spend longer periods in care, which raises questions regarding the welfare of such casualties. Poor triage equals poor welfare, as any wildlife casualty admitted into care will be stressed, not only as a result of its injuries, but also due to being in close proximity to people and unfamiliar surroundings. Such stress levels will not be conducive to recovery, and this must also be taken into account when considering the fate of casualties.

### Re-sighting and post-release survival

Survival based on ring recoveries of dead birds has always formed the foundation of studies for both rehabilitated and non-rehabilitated birds (Sharp 1996; Jessup 1998; Thompson 2013). Joys *et al.* (2003) analysed ring recoveries to investigate the effectiveness of rehabilitation in the UK and consistently found that most taxa or species of bird that underwent a period of time in captivity had a poor survival rate, however, without consistent and high coverage of re-sightings these results can be underestimates of survival. Prior to the research reported here, Thompson (2013) undertook survival research on both rehabilitated (reported in this research) and non-rehabilitated herring gulls born in the wild from south-west England, south Wales, and south-east England. In comparison to the 2,158 juvenile and nesting gulls ringed during this research, 2,940 juvenile and nestling herring gulls were ringed at the three wild sites over the same period, 1999 to 2010. Of these birds, 1,173 were individually sighted after ringing, with a maximum re-sighting of a single rehabilitated bird being 208, compared with only 39 of a rehabilitated bird. Rehabilitated juvenile herring gulls showed a lower median survival days (432 [± 36.9 SEM]) compared with the overall median from all three wild sites of (674 [± 63.5 SEM] days). However, when dividing the three wild sites separately, rehabilitated gulls survived better than those from the south-east of England (267 [± 67 SEM] days).

### Rehabilitation

Defining rehabilitation has always been contentious due to whether or not the focus is trained on issues related to animal welfare or it leans more toward conservation values (Aitken 2004). The action of taking in an injured or orphaned animal will automatically alter the animal’s behaviour and, in some circumstances, such as lack of predator avoidance, increase mortality and/or reduce the chance of survival back in the wild. Post-release monitoring remains the only method to measure the success or failure of intervention in natural- or anthropogenic-occurring events via wildlife rehabilitation.

Without the BTO ringing scheme, the primary aim of which is to measure the survival and dispersal of free-living birds (Baillie 2001), rehabilitators would be limited in the use of existing systems and procedures to ring rehabilitated birds. However, very few of the 800 wildlife centres in the UK (Mullineaux & Pawson 2024) undertake studies that involve post-release monitoring. This figure is on the increase with publications emphasising accountability of procedures and post-release survival (Mullineaux 2007; Kelly *et al.* 2008, 2012; Leighton *et al.* 2008; Murn & Hunt 2008; Cope *et al.* 2022; Yarnell *et al.* 2023; Mullineaux & Pawson 2024).

The question of whether rehabilitated animals can supplement failing populations has not been sufficiently explored. Herring gulls are not endangered, however, the numbers of the clade *L. argenteus* are declining in natural habitats (BirdLife International 2021) and herring gulls are a red-listed UK species, therefore should be considered a species of conservation importance (Stanbury *et al.* 2021). The origin of the decline is likely to be multifactorial, including the closure of landfill sites (Belant *et al.* 1998; Pais de Faria *et al.* 2021), increased rates of entanglement, and adult birds being affected by botulism and HPAI (Rock 2005; Soos & Wobeser 2006; Neimanis *et al.* 2007; Sonne *et al.* 2012; Pais de Faria *et al.* 2021; Verhagen *et al.* 2021; Lean *et al*. 2023). As in many city species, urban gull populations may act as a demographic reservoir for regional populations, with rehabilitation of all age classes helping support this reservoir.

### Animal welfare implications

Rehabilitation of wild animals provides an important link between our innate desire to care for the more vulnerable and the natural world. However, it is imperative that personal opinions and prejudices do not obscure our understanding of the factors required to provide humane and appropriate actions which benefit the individual animal. The subjective opinions of rehabilitators as to what constitutes humane treatment of an individual casualty can be difficult to quantify. UK Legislation such as the Animal Welfare Act (2006), if enforced, will safeguard against animal cruelty and suffering. Although the act applies primarily to domestic, farm and captive animals, it is essential that while wild animals are in captivity during their rehabilitation, key elements of the statute are adhered to, such as Prevention of harm (Unnecessary suffering; Section 4), Promotion of welfare (Duty of person responsible for animal to ensure welfare; Section 9) and Codes of practice (Section 14).

Understanding which factors contribute to the survival of wildlife casualties during rehabilitation and subsequent post-release survival was fundamental to this research. Concentrating on one single bird species has both advantages and disadvantages. On the one hand, for example, there are fewer confounding biotic variables, but there would be limitations in seeking to apply some of these results or recommendations to, for example, a terrestrial mammal. Nevertheless, the fundamental principles employed here are transferable to other bird taxa and should yield similar information.

Those who rehabilitate sick and injured wild animals will always need to justify their actions or continue to be scrutinised by those who believe that there to be no second chances in the natural world. Initiation of an open dialogue or forums within the fraternity of rehabilitators will improve techniques and procedures, potentially alleviating suffering. Post-release monitoring of animals is paramount if the treatment conducted and period of captivity are to be validated or reviewed.

## Supporting information

Thompson et al. supplementary material Thompson et al. supplementary material

## Supplementary material

The supplementary material for this article can be found at http://doi.org/10.1017/awf.2025.10047.
