# Supplementary material for: Reasons for admission and post-release survival of UK rehabilitated herring gulls (Larus argentatus) from 1999 to 2024
Source: Anim Welf. 2025 Oct 22;34:e69. doi: 10.1017/awf.2025.10047 (PMC12558741; doi:10.1017/awf.2025.10047)
Supplement: Thompson et al. supplementary material [file S096272862510047Xsup001.pdf]

RSPCA Wildlife Rehabilitation Protocol: Gulls

RSPCA

2013

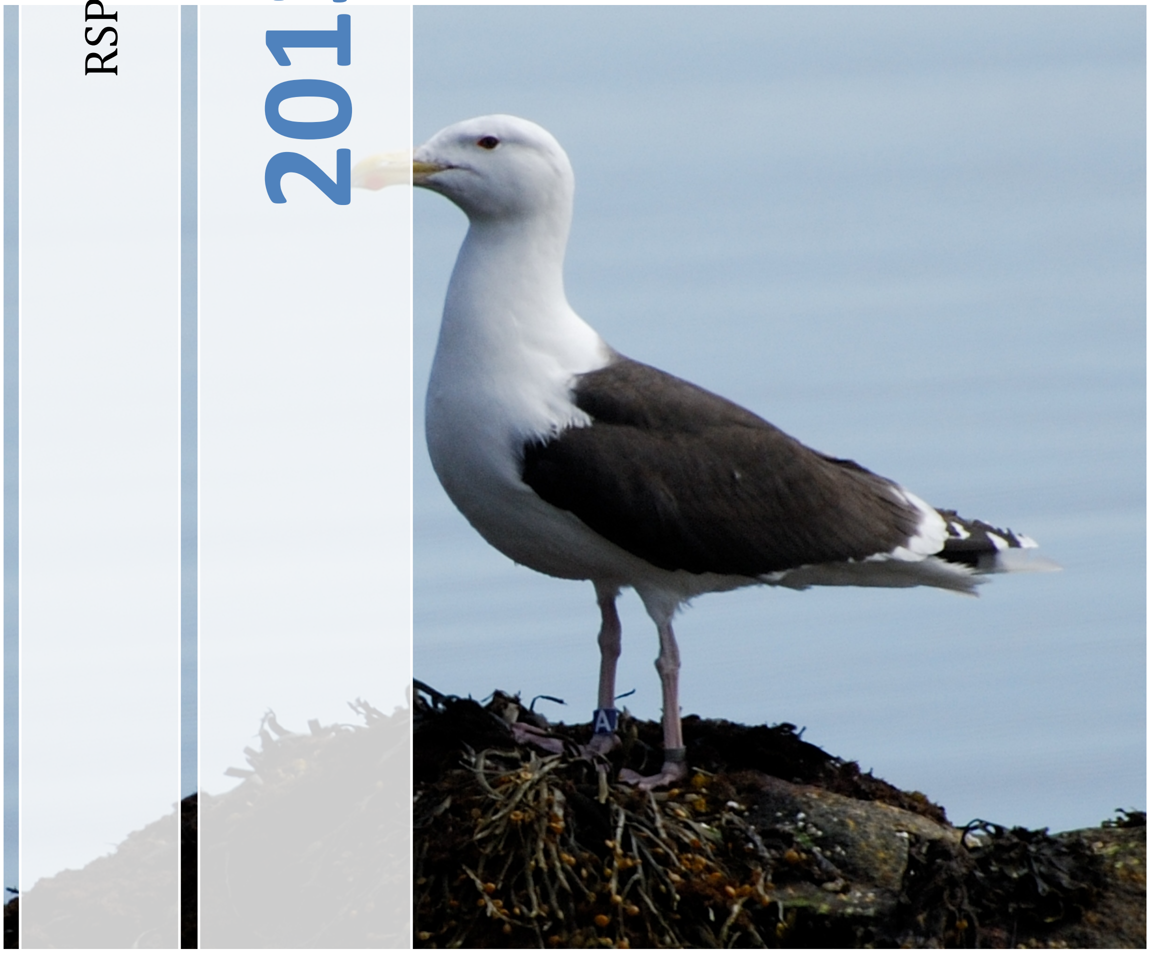

Photo: Adult Greater Black-Back Gull © Richard Thompson

© RSPCA

Wilberforce Way, Southwater, West Sussex,
RH13 0AE
wildlife@rspca.org.uk

Protocol for the rehabilitation of Gulls (family Laridae)

Contents:

1 Introduction 3
2 Species information 4
2.1 Species or group of species covered by this protocol 4
2.2 Identification of species covered by this protocol 4
2.3 General information on species 6
2.4 Importance of Environmental Enrichment 7
3 Pre-admission treatment. 9
3.1 Information should be collected on the following: 9
3.2 Advice related to care, e.g. diet, provision of heat etc. 9
3.3 Advice related to the treatment of particular problems. 9
3.4 Advice regarding the fitness of the animal for transport. 9
4 Health and Safety 10
4.1 Introduction 10
4.2 Risk assessments 10
5 Decision making – to treat or not to treat 11
5.1 Information should be collected on the following: 11
5.2 Triage 11
5.3 Treatment on admission 12
5.4 Flowchart 13
6 Housing 14
6.1 Indoor 1 (Intensive care) 14
6.2 Indoor 2 (Less intensive monitoring) 15
6.3 Outdoor 1 16
6.4 Outdoor 2 17
7 Diet 18
7.1 Food in the wild 18
7.2 Semi-natural captive diet 18
7.3 Artificial captive diet 18
7.4 Comments on feeding for all species 18
7.5 Supplements 19
7.6 Environmental enrichment 19
7.7 Notes on feather development 19
8 Preparation for release 20
8.1 Training the animal for survival 20
8.2 When is animal considered fit for release? 20
8.3 When to release 20
8.4 Where to release 20
8.5 How to release 21
8.6 Information 21
8.7 Marking requirements/tagging 21
9 Areas for research 21
10 Annexes 22
10.1 Glossary and abbreviations 22
10.2 Product details 22
10.3 Bibliography 23
10.4 References 23

Notes:
Areas highlighted within the text are areas that require further research or further clarification.

1 Introduction

The RSPCA's Wildlife Centres and the Wildlife Department have prepared a series of husbandry protocols for the different species that are admitted to the Wildlife Centres.

The protocols have been produced by amalgamating the working practices from each centre into one document which has then been discussed at a workshop before being agreed by RSPCA staff. Any areas where agreement cannot be reached are then highlighted as areas for future research.

Where possible, an expert (from outside the RSPCA) on the behaviour and ecology of the species in question was invited to attend these workshops so they could offer advice and comment.

These protocols are based on the experience and knowledge of our wildlife centre staff and are supported by research demonstrating their success. They are subject to review and updates will be added as and when required. New protocols will also be added over time.

This protocol contains general information only. The RSPCA makes no warranties, representations or undertakings about any of the content of the protocol (including without limitation any as to the quality, accuracy or fitness for any particular purpose of such content).

References in this protocol to any person or organisation do not represent an endorsement of that person or organisation, or its members, products or services.

To the extent permitted by law, the RSPCA does not accept liability for any loss arising out of or in connection with the use of this protocol.

Copyright notice:

The content of these pages is protected by copyright belonging to the RSPCA. You may download and copy the protocol to use only for the purposes of safeguarding animal welfare during rehabilitation but you must not sell or republish them. For any other purpose, you may quote a single paragraph of text from a page of the protocol without seeking our permission, provided that you acknowledge the RSPCA as the copyright owner of the material.

Pages or sections may be reproduced for teaching or study purposes without obtaining our prior consent. You may print and copy the pages for your private study or for teaching purposes in schools, colleges or universities provided in each case that:

1. copyright and source indications are also printed and copied
2. no modifications are made to the materials and they are not used as part of any other publication
3. the document is printed and copied entirely and is not used in a derogatory or misleading context
4. a maximum of 30 copies are made.

For any other publication of extracts from this protocol, please seek our permission. You can do this by emailing us at wildlife@rspca.org.uk

2 Species information

2.1 Species or group of species covered by this protocol

There are a number of species of gull that visit the UK during the year and that nest in the UK. They all come from one order, the Charadriifomes. There are a number of suborders again divided into further families. This protocol deals with the single family Laridae and divides them away from the skuas and terns. All the Laridae have three forward pointing toes that are webbed and feature a predominately white plumage; they are highly gregarious and frequent open sea, seacoasts, inland waterways and marshes. All nestlings of the Laridae family are semi-precocial, with eyes open, down covered, and stay in the nest until they are able to walk and where the parents feed them[i].

Family: **Laridae**
English name: Kittiwake (or black legged kittiwake)
Latin name: Rissa tridactyla

English name : Black-headed gull (or common black-headed gull)
Latin name: Larus ridibundus

English name: Common gull (or mew gull)
Latin name: Larus canus

English name: Lesser black-backed gull
Latin name: Larus fuscus

English name: Herring gull
Latin name: *Larus argentatus*

English name: Great Black-backed gull
Latin name: Larus marinus

Other species encountered in RSPCA wildlife centres to date include the yellow-legged gull, *Larus michahellis*.

Names in brackets indicate the full name according to the current British Ornithological Union's British bird list[ii].

2.2 Identification of species covered by this protocol

2.2.1 Adult

The body plumage of all British breeding gulls dealt with here is white with a grey or dark grey mantle, and upper wings; in winter, the tail in the adults of all these species remains white having no black band across the end as immature birds show. The amount of black on the primary feathers varies between species, as does the colour of the feet and legs.

Recently a number of subspecies of the gulls have been separated out into species of their own[ii] notably the yellow-legged gull *Larus michahellis* and Caspian gull *L. cachinnans* from the herring gull thus creating some confusion. A further complication may be the fact that certain species interbreed notably black-headed and Mediterranean gull *(Larus melanocephalus)* and the herring and lesser black-backed and great black-backed gulls and thus will show characteristics of both. Occasional misidentification may therefore occur.

© RSPCA Gulls 2013.docx

Kittiwake
Black legs and clear yellow bill is a clear feature of these gulls. Wing-tips are wholly black without "windows" of white. In winter the normally white head may develop black crescent behind the eye and around the ear.

Black-headed gull
The red legs and red bill of this gull are unmistakable. In addition it is the only European gull with, in adult plumage, a brown hood in summer a white partial eye ring may be present, in winter this diminishes to small, often absent, flecked bands across the head and noticeable blackish ear spot. The iris is brown and the orbital ring is red

Common gull
Yellow-green bill and legs distinguish this gull. The head colour has a dusky hue to it. The wing tips have white "mirrors" although these vary in size with the seasons being smaller in winter. The iris is brown and the eyes have a red orbital ring.

Lesser black-backed gull
The bill is yellow with a red spot near the gonys on the lower mandible and the legs are yellow. The mantle is darker grey (often described as "slate grey") in this species and may often appear almost black in some lighting situations. The iris is pale yellow and the eye has a red orbital ring.

Herring gull
The bill is yellow with a red spot near the gonys on the lower mandible and the legs are pink. The mantle is a light grey. The iris is pale yellow and the eye has a red orbital ring.

Great black-backed gull
The deep and heavy-looking bill is yellow with a red spot near the gonys on the lower mandible and the legs are pink. However differentiating it from the previous two species is its larger size and almost black mantle. The wing tips are usually all white except for the occasional black spot.

2.2.2 Young

All very young *Larus* gulls are semi-precocial staying in the nest until they can walk easily. All are down covered coloured olive green to soft buff-brown with dark almost black spots. All have dark or black beaks except the black-headed gull nestling that has a pink beak with a dark spot on the tip. The kittiwake is much paler than all the others – almost white underneath to a soft light grey on the back.

Kittiwake
A colonial cliff nester, although the large warehouse buildings on Tyneside hold comparatively small populations.

Black-headed gull
Probably the UK's most inland gull nesting in often very large colonies. A ground nester utilising islands and low lying ground – may be subject to loss of nests through flash floods.

Common gull
Often found in the company of other nesting gulls but rarely forms large colonies on its own. Almost exclusively confined to Scotland although small groups nest on the south and East Anglia coasts. A ground nester utilising sites such as coastal islands and stacks, islands in freshwater and isolated moorland hills as well as some roofs and protected industrial sites.

Lesser black-backed gull
In the wild it is almost exclusively coastal (where it prefers to nest amongst vegetation in gently undulating dunes) but some isolated colonies occur inland, for example, Tambrook Fell. Several wild colonies decreasing (pairs at Orfordness decreasing from 25,000 in 1998 to 5,000 in 2007). Since the mid-1980s this species has increased dramatically in urban colonies, especially in the Severn Estuary Region.

Herring gull
Has declined by more than 40% in the last 25 years along our coasts. In wild colonies tends to prefer cliffs and craggy areas for nesting and this preference is echoed in towns where complex roof structures are frequently utilised. In contrast to wild colonies this species is faring well on urban rooftops in many towns throughout Britain and Ireland.

Immature Birds
Most of the *Larus* gulls take a number of years to completely moult into their adult plumage (up to four years in the great black-backed gull) so the plumage can be used to age these birds. However, experience is required and the subtleties of plumage colour and its distribution are difficult to describe in this work. Using good reference works[iii] will provide a means of ageing (and identifying) most of these young birds.

2.3 General information on species

Kittiwake
- As a pelagic species fish and marine invertebrates make up the bulk of its diet.
- Not often seen feeding inland unless storm-blown. Feeding method is by taking from the surface (dipping) and also some scavenging especially from fishing vessels both at sea and in port.
- A very social species during the breeding season but such aggregations break up during the non-breeding period and birds form small mixed groups with other gulls but may equally be seen as solitary individuals.
- Rarely seen far from the sea.

Black-headed gull
- Insects and other invertebrates make up the bulk of the diet but also household waste, and plant material. A wide range of foodstuffs can be taken according to locality and season.
- A social species at all times of the year.
- Feeding method varies according to prey but capable of a range of strategies from following the plough to foot paddling and even plunge diving from 2 metres above water. Fond of swarming ants and chafers, and may take fruit by hovering over fruit trees. Piracy is also known in some birds.
- Frequently seen far from the sea often on farmland but will also be found at popular feeding areas such as public parks and on sports fields feeding on invertebrates.

Common gull
- Mainly terrestrial invertebrates that tend to be foraged from drier ground than the black-headed gull. Will also take fish, scavenge often through piracy and follow the plough.
- May form large groups out of the breeding season and also becomes part of aggregations of other species of gull.
- Equally at home near the sea or inland although rarely strays far out to sea from the coastal fringes.
- When young can fly they often fly straight to grass and arable areas. But will as easily fly to estuaries and sand shores.

© RSPCA Gulls 2013.docx

Lesser black-backed gull

- Largely omnivorous including small vertebrates and invertebrates, but flocks will be seen feeding over large shoals of fish at sea; plant material and also scavenged material from waste tips is taken. Often seen feeding in flock of hundreds. Feeding methods include taking from the surface and plunge diving.
- Very social during breeding season out of this season will be seen in groups including other species of gull notably the herring and great black-backed. Appears to be more gregarious outside the breeding season than the great black-backed or the herring gull.
- Habitat throughout the year is variable including waterways, estuaries, as well as sports fields, rubbish tips and ploughed fields, landfill and ploughed fields.

Herring gull

- Another omnivorous species taking a very wide range of food. Often seen following a range of seagoing vessels for scraps particularly the waste from fishing boats. May be seen in large mixed and single species flocks at waste tips scavenging for food. A wide range of feeding methods are used some may be learned from conspecifics.
- May be found almost anywhere where there is adequate and suitable food. Will however, still follow ships and other sea-going vessels. Large flocks may congregate in favoured area including landfill sites.

Great black-backed gull

- Voracious predator, scavenger and pirate of a wide range of food. Known to take mammals, birds and invertebrates from in flight eg grabbing puffins arriving at nesting cliffs, to opportunistic "dive-bombing" flights into groups of nesting birds or feeding rabbits. Small mammals are eaten whole but will tear prey apart rather like a bird of prey.
- May be seen on its own, in small groups or in the company of other larger gull species. Often large aggregations occur where there is good feeding.
- Unlike the other Larus gulls may be seen some significant distance from shore out to sea (up to 150km) but availability of access to urban areas and human refuse provides adequate feeding and shelter.

2.4 Importance of Environmental Enrichment

Enrichment Suggestions

- When creched, juveniles enjoy cardboard boxes on their side or blue fluted boxes to rest in and sit on top of.
- Provide a variety of food for herring and lesser black-backed chicks. For example day-old-chicks and cooked chicken carcasses. A regular change in food items adds to enrichment - a diet of one food item can lead to boredom. Provide mealworms for common and black headed gulls.
- Being a colonial species small groups of the Larus species "do better". Ensure each group is formed of similarly aged individuals and keep groups small (up to five). Keep species together and keep chicks and adults apart unless they are from the same family group. Keep adult gull species apart and never put adult great black-backed gulls in with other gull species.
- These birds are regular bathers in fresh water. Fresh bathing water can be provided to chicks when the contour feathers have come through on chest and back.
- A variety of perches and blocks will provide enrichment. Dominant birds will always select high perches.

NOTE: Avoid yellow colour-rings on legs of chicks especially those of herring and lesser black-backed gulls.

© RSPCA Gulls 2013.docx

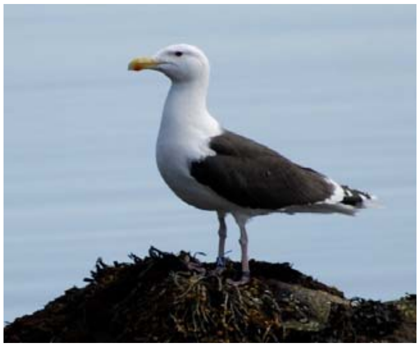

Figure 1: Great black-backed gull

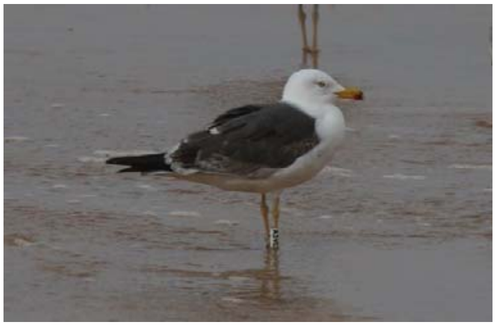

Figure 2: Lesser black-backed gull

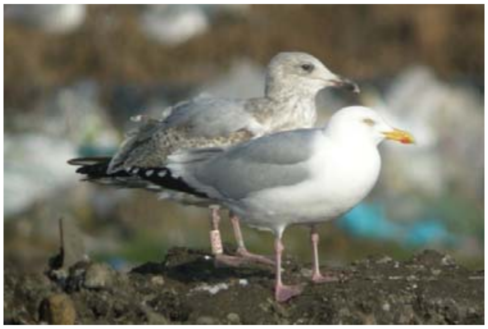

Figure 3: Herring gull (adult and immature)

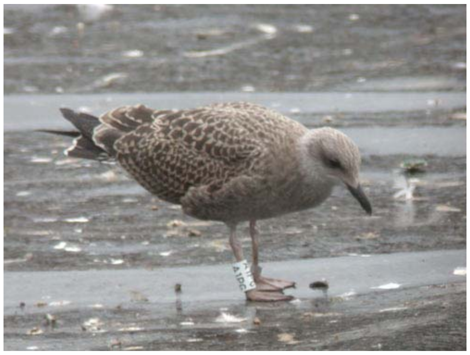

Figure 4: Immature lesser black-backed gull

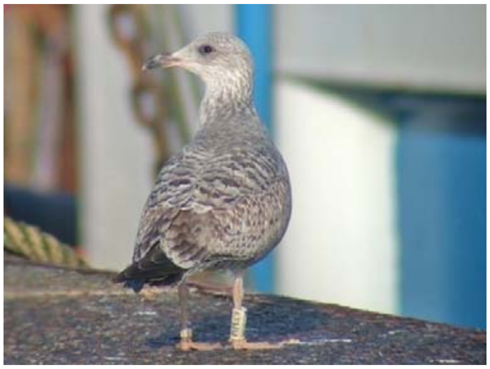

Figure 5: Herring gull first year

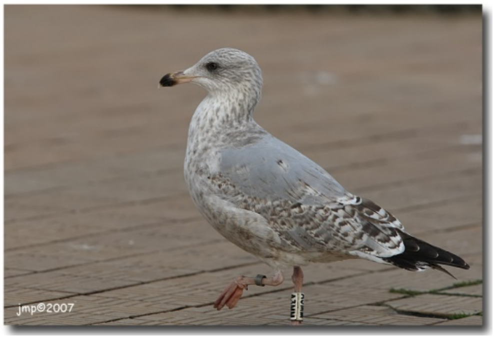

Figure 6: Herring gull second year

3 Pre-admission treatment.

This part of the protocol is to provide information for telephone queries regarding gulls and their rehabilitation, prior to receiving a gull at an RSPCA Wildlife Centre. There are two possible scenarios:

A member of the public is reporting a sick/injured or orphaned gull and wants further information as to what to do.
Prior to admission, some animals may be held at a veterinary surgery or other facility. Some, if not all, of these facilities may request information on care of the animal, before they send it to an RSPCA centre.

Does the gull need to be admitted? Try to determine if the bird needs treatment, if it can be treated on site or left alone?

3.1 Information should be collected on the following:

a) Species (often a finding location helps with identification),
b) Extent of injuries, evidence of shock,
c) Body condition, any previous injuries,
d) Age of animal, dependent (non-flying) young (speckled plumage) normally able to fly or breeding adult (basic white plumage),
e) Location animal was found (important to ensure adults are returned to the same area, however, see section 6.4 on page 20),
f) All records of previous treatment (if from another establishment).

3.2 Advice related to care, e.g. diet, provision of heat etc.

- Collapsed, sick or injured birds require heat but avoid heat where botulism is suspected.
- Don’t feed before transport

3.3 Advice related to the treatment of particular problems.

- All birds should be isolated to avoid fighting small chicks may be kept together.
- Recumbent animals need water and food bowls very close and regular cloacal washes to avoid heavy soiling.

3.4 Advice regarding the fitness of the animal for transport.

- Single adults and small groups of young are transported in a standard pet carrier of 30cm x 45cm x 25cm, but cover wire slots and doors with towel or similar. Do not use wire cages.
- Watch for heat build-up in boxes containing a number of chicks.
- Ensure boxes are sound when carrying adults.

4 Health and Safety

4.1 Introduction

The RSPCA has developed the Wildlife Centre Protocols to provide guidance and advice on the keeping of certain species of wild animal for rehabilitation. Anybody who intends to treat sick, injured and/or orphaned wild animals must accept that there are risks in doing so. Some wild animals are potentially dangerous and may be capable of causing serious injury. Furthermore, all wild animals have the potential to carry parasites, disease and bacterial infections. Some of these may be passed to humans (zoonoses) or to other animals, either domestic or wild. Barrier nursing methods should be used to minimise the spread of these infections between animals.

4.2 Risk assessments

It is recommended that any establishment admitting gulls should complete risk assessments for all areas.

This is a brief summary of some of the possible risks and suggested ways to reduce the effects.

Members of public are advised to use gloves or a suitable alternative (e.g. towel) when handling gulls and to keep dogs etc away from injured wildlife.

Hazards	Control measures	Level of risk
Bites and scratches	Gloves to be used when restraining	Low
Diseases/Zoonoses<br>Campylobactor	Gloves should be worn when handling and wash hands after handling<br>Face masks when washing down enclosures and handling young birds.<br>Treatment areas must be cleaned thoroughly after examination	Low
Parasites	Gloves should be worn when handling	Low

© RSPCA Gulls 2013.docx

5 Decision making – to treat or not to treat

5.1 Information should be collected on the following:

A range of information is required to make the most appropriate decision for the animal in care. Information collected under 3.1 on page 9 will be used to make an assessment, as will observations of the bird itself. A veterinary opinion will be taken into full account where necessary.

5.2 Triage

5.2.1 Assessment relevant to the condition of the animal

Options for the animal are: euthanasia, treatment or immediate return to the wild. The considerations listed below will help to guide this decision as many of these conditions indicate a poor survival to release.

Call the RSPCA Wildlife Centres for further advice.

Table 1: Conditions that normally indicate euthanasia (see notes below)

Severely and obviously broken wings	PTS
Obviously broken legs	PTS
Broken coracoid (usually identified on x-ray)	PTS
Two limbs missing	PTS
Birds with freshly missing leg	PTS[1]
Orphans with severe secondary problems	PTS[2]
Oiled birds with other problems	PTS
Old breaks	PTS but see notes
Birds showing signs of angel wing	PTS[3]
Any deformity, most often of the beak	PTS
Blind in one or both eyes	PTS

- Euthanasia may also be considered where the bird is unable to lift its head and/or grip firmly with the beak due to severe weakness. Gulls suffering from these symptoms are usually recommended for PTS but some success has been achieved with these cases. These symptoms may indicate the bird is suffering from botulism.
- It should be noted that as these birds are (mostly) large and robust many minor injuries can be treated with success.

5.2.2 Assessment relevant to the centre and the management of the animals

- Is an experienced vet, Wildlife Assistant or wildlife centre supervisor available to see the animal within an appropriate time-scale?
- Is suitable housing/space available to accommodate the animal according to this protocol?

[1] Birds admitted with one leg and which have survived well with that one leg may sometimes be rehabilitated. Full details of the reasons for admission and site of finding must accompany the bird, as this can be essential to the decision-making process.
[2] Minor injuries may be treated in certain individuals.
[3] Although noted as a condition for PTS it is rarely seen on admission. Carpal wing problems in chicks are possibly due to intraspecific conflict at nest site.

- Are current staffing levels sufficient to give the bird(s) the time required for good rehabilitation?
- What is the predicted intake of animals in the short term?
- Admission numbers will be controlled carefully to avoid overcrowding. Bottlenecks occur when high admissions of small chicks coincide with high incidence of botulism in adults
- A good supply of quality fish must be assured.

5.3 Treatment on admission

5.3.1 If any of the following are identified proceed to vet examination.

- Refer all conditions except apparently healthy chicks and juveniles on maiden flights that may just be “grounded”.
- X-ray all grounded adult birds that do not appear to have symptoms of botulism. This is particularly important where birds are likely to have been shot.

5.3.2 If none of the problems listed in 5.3.1 are identified:

- Place individual ID ring on every admission. Can be plastic or aluminium but avoid confusion with British Trust for Ornithology (BTO) rings. Use any colour except yellow or red rings as these may cause minor aggression between individuals.
- The use of biometrics is a good way of assessing where chicks and fledglings should enter the system. (See information in section 4 on page 14)
- Measure the wing chord and weigh the bird recording both pieces of information.

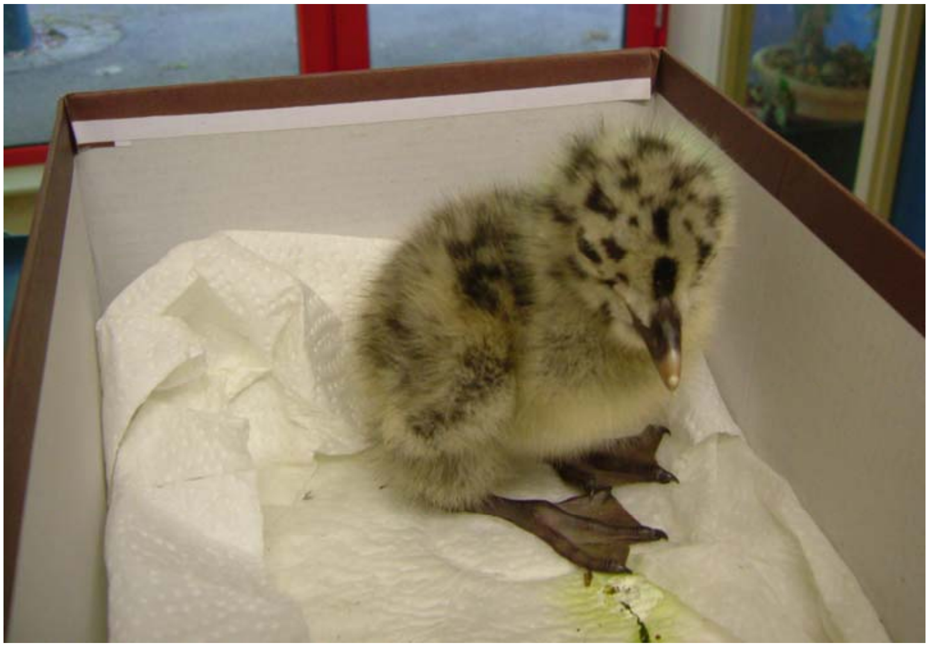

Herring gull chick on arrival (note the egg tooth of this very young chick)

5.4 Flowchart

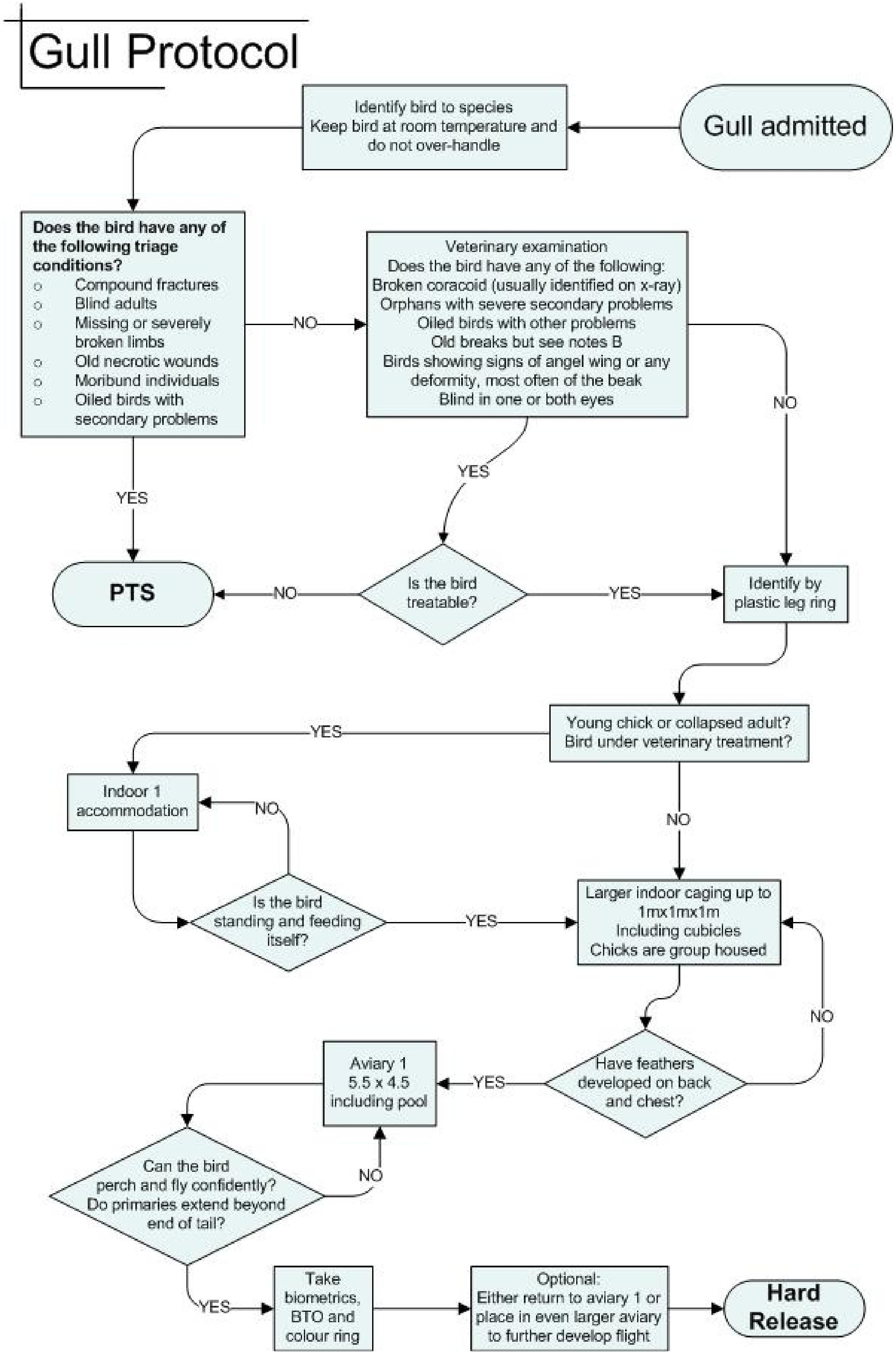

6 Housing

The progression from *Indoor1* to *Indoor 2* to *Outdoor 1* to *Outdoor 2* represents the movement of an animal through the Centre as its condition improves. Not all of the categories will be applicable to all these species, their condition etc. The need for environmental enrichment should be considered and identified wherever possible for each of the following sections. Enclosure sizes are all described as length x width x height.

6.1 Indoor 1 (Intensive care)

Enclosure
Avoid any accommodation with bars or wire mesh for birds of any age. If door has bars, blinds (e.g. towels placed on the inside of the bars) are used to prevent problems such as damage to the forehead and primary and tail feather breaks and abrasions.

Use a suitable plastic or cardboard box/pet carrier 30 x 45 x 25 cm for:
- Sick, collapsed and injured birds and
- Small chicks under 200 – 250gms.

Chicks can be creched with animals of similar age from admittance in container described above in groups of 2 – 3. Monitor to make sure all birds are feeding and there is no bullying.

Larger accommodation is used for older birds and those that can stand:

Young	60 x 40 x 45 cm
Adult	70 x 40 x 45 cm
Great black-backed gull	71 x 71 x 71 cm

Table 2: Accommodation sizes

Sick, collapsed, cold or thin adults or newly hatched chicks will be given a heat pad placed to the side of the container – this is particularly important for single birds.

Keep great black-backed gulls separate.

Lighting
- Room lighting and normal daylight hours sufficient

Substrate
- Layers of newspaper that can be removed regularly to avoid heavy faecal build-up.
- Towels placed over thick newspaper can be used for recumbent adults, immature birds and large juveniles.

Temperature
- Chicks and sick, emaciated and lethargic young birds and adults should have heat provided. *Vet-bed* including a heated pad for additional heat – this is usually placed alongside of the container.
- Feathered young and injured but bright adults can be kept at room temperature.
- Waterlogged juveniles (usually wet following their maiden flight) will require carefully monitored heat for revival.
- For notes on the thermoregulation of young see O'Connor 1984[iv]

Ventilation
- Ensure good ventilation at all times.

Access to Water

- Water for chicks provided only through moist fresh fish provided in small shallow bowls. Do not provide a separate water bowl – this will prevent chicks sitting in water and becoming wet and chilled.
- Adults and immatures are provided with fresh water in a 18cm diameter steel non-spill bowl.

Environmental Enrichment

- For chicks companionship is essential.
- Always match chick weight for weight. Smaller chicks can be mixed with larger but may require feeding for tweezers until seen feeding for themselves..
- For adults in this accommodation they will be sick and due to the possibility of limited space will rarely be provided with additional enrichment (other than food and water). BUT move these birds on as soon as possible.

6.1.1 When to move to next stage:

Chicks:

- Herring and lesser black backed gull chicks should weigh between 200 & 250gms and/or be self-feeding.

Adults and immatures:

- These should be standing confidently and be self-feeding.

6.2 Indoor 2 (Less intensive monitoring)

Enclosure

- Pen or cubicle 2.5 x 1.5 x 2.5m with the ability to take some water facility – see enrichment, later.
- Herring and lesser black-backed gulls are moved into larger areas such as open-topped bays or larger cubicles up to and greater than 2.5 x 1.5m.
- Birds can be creched together.
- Continue to avoid overcrowding and watch for bullies isolating as necessary.
- Adults and immature birds with limited flight and that are still being monitored (eg weight build-up or confirmation of flight) can be moved into a cubicle.
- In these enclosures they will usually stay calm but at early signs of stress birds should be moved on to next stage of accommodation.

Lighting

- Room lighting following normal daylight hours is sufficient.

Substrate

- These areas all have a concrete base incorporating a drain.
- For all ages soft flooring to prevent sores is used: blankets, sheets, *Astroturf*, large towels, rubber mats are all placed over thick newspaper. WARNING: rubber mats may be pecked and destroyed - be vigilant against swallowing.
- Chicks will benefit from the provision of ledges (see environmental enrichment).
- Adults can be provided with a further covering of newspaper to reduce excessive soiling of their plumage and to help maintaining hygiene.
- The production of large amounts of faecal matter during this stage presents a cleaning challenge but regular attention to newspaper changes and “squeegee-ing” areas will keep areas clean.

Ventilation

- Needs to be good.

Access to water

- Provide a pool or large 80 x 30 x 10cm deep water containers to bathe in and encourage preening.
- Introduce water gradually ie just enough to paddle in at this stage start with only 25mm and build up.

- Chicks indoors will be provided with small drinking containers to discourage bathing.

Environmental Enrichment
- Companionship is important but watch for bullying. Isolate bullies as necessary.
- Providing perches in the form of "*Thermalite*" or concrete blocks 45 x 25 x 10cms or cardboard boxes gives access to height. These may reduce contamination of large areas by concentrating droppings in a smaller area.
- Cardboard boxes also provide access to a material that can be pecked and destroyed. This may also reduce aggression to other gulls in care.
- As the birds' feathers develop more enrichment can be provided dropping food items such as day-old chicks for additional enrichment.
- Change enrichment frequently and at irregular intervals to avoid boredom.

6.2.1 When to move to next stage:
- All birds should be eating well on their own (established by observations) active, waterproof and beginning to make flight.
- Chicks will be developing contour feathers on back and chest. Probably no primaries but may have a little growth to the tail. Herring, lesser black-backed and greater black-backed gulls chicks will still have downy, brown spotty head.
- Weighing will be required for birds under par and/or those that are still on treatment.
- Adults and immature birds can go from here to outdoor 2.

6.3 Outdoor 1

Enclosure
- At this stage it is vital that birds have access to clean water to bathe and clean their plumage.
- An enclosure sized 5.5 x 4.5 x 2.5m with sides and roof netted. Sides should be solid for 1 metre from the ground.
- An aviary with a pool is ideal. Ample space for standing must be provided although concrete sides need to be covered with softer material to prevent foot damage. The water will need to be changed twice a day. If no pool is available, large dog beds filled with water can be used. The water will need changing 4 to 5 times a day in this instance.
- A ledge placed off the ground provides high perching.

Substrate
- Concrete base incorporating a drain for easy cleaning.
- Rubber mats or *Astroturf* to protect feet.

Shelter
- Solid side protection should provide sufficient shelter and prevent feathers and beaks poking through mesh. Should not need covered areas, as birds should be waterproof.
- Any waterlogged birds at this stage need further investigation.
- In really bad weather or for vulnerable individuals half *Vari-kennels* or a covered area of the aviary may provide some shelter.

Access to Water
- Provide pool or large container of water preferably with graduated sides.
- Water should be deep enough for bathing and swimming 15 – 20cms

Environmental Enrichment:

At this stage water for chicks, juveniles and adults is essential and for some will provide enough enrichment on its own but any further activity will always be welcome.

Chicks and juveniles
- The young will investigate all things in the aviary; feathers, twigs, leaves, stones – anything!
- Thick straight branches and chunky blocks to perch on, to peck at and investigate.
- Cardboard boxes are used for shelter and for pecking to destruction.
- Companionship and interaction with other gulls.

- Provision of dead day-old chicks at irregular intervals will provide enrichment as the birds pull and tear the food apart.

Adults

- Little enrichment is required but perches - logs, ledges and blocks – provide flight opportunities and perching.

6.3.1 When to move to next stage:

- Chicks – now juveniles - will have only contour feathers and primaries that will extend past the end of the tail.

6.4 Outdoor 2

Enclosures

- Larger enclosures dominated by water areas of approximately 7m x 5m are suitable. Ledges are provided for standing and feeding.

Substrate

- Rubber matting distributed around pool sides or where appropriate grass sides.
- However, if outside paddocks are used natural turf and earth is quite appropriate.

Shelter

- These enclosures are almost exclusively used during the summer when admissions are at the highest. Very little shelter. Birds have been assessed based on their ability to thermoregulate and their plumage should repel even the heaviest downpour.

Access to water

- Lots of water are needed at this stage. Water is changed up to twice a day where large numbers are housed. At all times, the ability to maintain a reasonable level of hygiene is paramount. The careful management of faeces in this area is essential.

Environmental enrichment

- The pools provide bathing and perching opportunities. Essentially an area for pre-release assessment.
- Enrichment is predominately through foraging where the birds may find natural twigs, leaves and stones.

6.4.1 When to move to next stage:

The next stage is release. See section 8.2 on page 20.

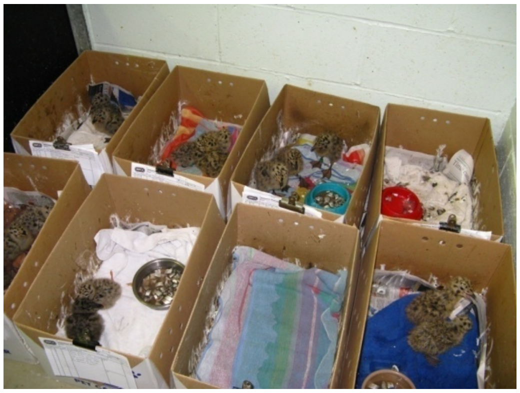

Figure 7: Boxes used to house large numbers of young gull chicks at Mallydams Wood.

© RSPCA Gulls 2013.docx

7 Diet

7.1 Food in the wild

For both adults and young see above under section 2.3 on page 6.

7.2 Semi-natural captive diet

Table 3: Adult diet in captivity

Species	Food type	Amount/frequency
Herring, lesser and greater black-backed gulls	Sprats<br>Day-old chick<br>Whitebait<br>sandeels	Feed *ad lib* but usually the equivalent to between 10 and 20 sprats per bird per feed.<br><br>1 day old chick daily per bird<br><br>Give all foods fresh twice daily, but top up food bowls regularly if eating all food. Food can also be scattered around the enclosure.
Kittiwake		
Black-headed gull and common gull	Mealworms can be provided in addition to the above.	

Table 4: Chick diet in captivity

Species	Weight range	Food type	Amount/frequency
Herring, lesser and greater black-backed gulls weighing between approximately:	60gms-120gms	Finely chopped sprats or whitebait.	Large amounts offered in enclosures to avoid competition.<br><br>Very young chicks are primed 3-4 times daily, until self-feeding.<br><br>Once self-feeding food is replenished 3-4 times daily.<br><br>Food can also be scattered around the enclosure.
	120gms – 250gms	chopped sprats	
	250gms – 600gms	Roughly chopped and whole sprats offered simultaneously	
	Over 600gms	All sprats whole, rough chopped herring or mackerel and a daily day old chick provided whole.	
Kittiwake		Whitebait in initial stages then whole sprats	
Black-headed gull and common gull		Whitebait & some insects	

7.3 Artificial captive diet

Adults & Young

- **No canned domestic animal foods are to be offered.**
- Fish is the preferred diet through all stages and will promote good quality growth.

7.4 Comments on feeding for all species

- <u>No liquidized food is administered</u>.
- Birds are encouraged to self-feed on whole or chopped fish. Severely inappetent animals will be blood tested and x-rayed by an experienced vet to investigate for possible obstructions or other problems. If the bird remains inappetent something is wrong and needs investigating.

© RSPCA Gulls 2013.docx

- Critical care patients may be tube-fed *Zoolyte* or *Lectade* at the rate of 10% of body weight divided into 4 equal doses in 24 hours, progressing to AD and *Zoolyte* after the first 24 hours.
- Providing some mealworms may encourage eating.

7.5 Supplements

7.6 Environmental enrichment

- The provision of a variety of food items that the birds can manipulate and investigate and which does not involve pet food or vegetables is among the best enrichment that can be provided.

7.7 Notes on feather development

7.7.1 Feather quality

Both poor quality feathers and fret marks may be caused by deficiencies in diet, stress or both. Work on birds of prey and species of passerine bird have shown that poor diet during the growth of the feathers, either while the bird was in the nest or during normal moult, can cause weak feathers and poor plumage. It may lack lustre and iridescence, the colour may be poor and there may be a general dishevelled look to the bird. The feathers may feel dry and "straw-like" and the feather edges look worn and tatty. The plumage may also contain broken and bent feathers.

Poor feather quality may mean that flight may be severely affected or impossible. The plumage may also not be waterproof and so may result in the bird being unable to maintain body temperature.

7.7.2 Fret marks

Fret marks show in feathers as lines across the vane; they may also show as ragged breaks, splits and "cuts" in the edges of the feather - see photograph below. These abnormalities are caused by inadequacies in the diet while the feather is growing. The result may be a significant flaw in the feather frequently leading to breaks across the line of weakness. These conditions are of particular concern when found in one or more of the following feather groups; primaries, secondaries or tail feathers.

7.7.3 Importance of diet

Poor feather quality is a problem that can be avoided by providing a proper diet. It is therefore important to follow a good quality dietary regime such as that outlined above. Failure to do this can result in birds having to be kept for extended periods as they would not be fit for release at the correct time, or possibly euthanasia if the damage to the feathers is too extensive.

© RSPCA Gulls 2013.docx

8 Preparation for release

8.1 Training the animal for survival

If birds have been "prepared" using the techniques and advice given above additional "training" may not be required.

8.2 When is animal considered fit for release?

Adults

- Maintaining good plumage by bathing daily.
- Flying well, weatherproof, no sore legions on feet, flight feathers in good condition.
- Adults become agitated, flying frequently and dominating high perches.
- Feeding may drop and with botulism cases, no further green faeces will be evident.

Juveniles

- All of the above.
- Chicks ready to go may eat less food and spend more time swimming than on the side.
- Chicks will drop weight prior to fledging so chicks may not reach the weights above.
- A wing chord of about 360mm in herring and lesser black-backed gulls is a good indicator the bird is fully fledged.
- Great black-backed gull chicks have a much slower growth rate, so identify these early.

NOTE:
Good body condition score is important, as size may be quite variable in juveniles.

8.3 When to release

- A morning release is best.
- During settled conditions.
- On a receding tide if coastal release.

8.4 Where to release

Adults

- These are released back where they were found. If this is not suitable use the release criteria for juveniles.
- From current ring recovery evidence a release from the rehabilitation centre is appropriate for fully adult herring, lesser black-backed or great black-backed gulls if the site of finding is less than 50 km distant from the centre.
- However release from coastal sites that do not have high density of nesting birds may be preferable. Ideally they should not be released inland unless at a landfill site during opening hours. (ring recovery evidence).
- Black-headed gulls are generally admitted out of the breeding season. Release at sites of known gull concentrations. The UK has large numbers of eastern European birds during the winter so birds could easily be part of this population. (Ring recovery evidence and controls)
- Common gull - Very few breed in southern England the birds found here are therefore mostly wintering population. Release into known common gull roosts and feeding areas.
- Kittiwakes. Always release at coastal areas.

Juveniles

- Herring, lesser- black-backed and great black-backed gulls are released at the coast, ideally at a site with a large tidal reach including rock pools and mudflats.
- Black-headed gull – ideal release will be at an inland water body if the birds are known to occur at these sites. Ideally release with other gulls of the same species.

- Common gull – These birds have a very localised distribution. There is a need to know the breeding distribution or regular occurrences at chosen site of release.

8.5 How to release

- A hard release is ideal for all gull species.
- Adults
  - ✓ Can be released singly or as part of a group.
- Immature
  - ✓ Ideally, immature, artificially reared chicks are released as a group.
  - ✓ Large groups can be successfully released (12 or more birds).

8.6 Information

The following measurements should be taken prior to release.

- Age
- Weight
- Bill depth & total head & maximum wing chord.
- Always check species has been identified correctly before ringing, as confusion between species is possible.

8.7 Marking requirements/tagging

- All gulls should be marked with authorised rings from the BTO. Colour rings can be used to track individual birds. Both activities are undertaken under license from the BTO.
- Ensure any temporary rings and other identification marks used by the centre are removed before release.

9 Areas for research

Areas that need further research include:

- Chick growth and diet coupled with food assessment at the nest.
- Monitoring the survival of gulls that have been injured by air guns.
- Breeding dynamics of adult urban nesting birds and cost of rehabilitation during breeding season.
- Further research on whether it is always best to take adults back to where they are found.
- Is chick survival affected by time in care?
- Is there a way to establish age from tarsus length?

10 Annexes

10.1 Glossary and abbreviations

**Adult**	A bird in full adult plumage. See Grant (1982)[iii]
**Biometrics**	Measurements taken to provide greater detail on the biology of birds. Data includes: plumage, size(s) and condition. (Further detail can be found in the *Ringers' Manual*[v]. C/f: wing chord, gonys, tarsus etc.
**Botulism**	A toxin (*Clostridium botulinum*) that affects the muscles of a bird causing paralysis. Usually reported in gulls that have been feeding on rubbish tips.
**BTO**	British Trust for Ornithology
**Chick**	A young bird that is not walking well and would still be within the nest.
**Contour feathers**	The basic feathers of a bird.
**Coracoid**	The bone that is paired and forms the bridge between the sternum (breastbone) and the shoulder. (Behind the "wish-bone" on a roast chicken.)
**Gonys**	The midline of the lower mandible from the tip to the back.
**Hard release**	A method of release whereby the animal is released without support feeding or provision of additional shelter.
**Immature**	A young bird that has left the nest but has not yet got its adult plumage.
**Intraspecific**	Within the same species.
**Juvenile**	A young bird that has left the nest but is not yet flying and is still nominally within the nest area.
**Pelagic**	Inhabiting the open seas.
**Precocial**	A chick hatched covered in down and leaves the nest soon after hatching and does not necessarily return.
**Primary feathers (primaries)**	Major flight feathers – usually the longest feathers on a bird's wing.
**Semi-precocial**	A chick hatched covered in down and is capable of leaving the nest soon after hatching but stays in, or very close to the nest.
**Soft release**	A method of release whereby an animal is released with support food and/or shelter prior to attaining its total freedom.
**Tarsus**	Properly the tibiotarsus – the lower part of a bird's leg before the foot.
**Wing chord**	The length of the wing measured from the carpal joint to the tip of the longest primary feather. (See BTO *Ringers' Manual*[v].)

10.2 Product details

**Aquavits**	A vitamin and mineral supplement for fish-eating animals.<br>*International Zoo Veterinary Group, Keighly, N Yorkshire, UK*
**Astroturf**	A brand of artificial turf. Usually made from plastic and rubber with additional fibres for realism.
**Thermalite**	An aircrete building block.<br>Hanson Limited. www.hanson.co.uk.
**Sky /Vari-kennels**	Pet carriers moulded in durable plastic with wire side mesh and doors. Can be dismantled into two halves.
**Vet-bed**	A proprietary pet bedding.<br>*Petlife International Limited, Bury St Edmunds, Suffolk*<br>www.petlifeonline.co.uk
**Zoolyte**	A water soluble oral rehydration and probiotic supplement.<br>*International Zoo Veterinary Group, Keighly, N Yorkshire, UK*

© RSPCA Gulls 2013.docx

10.3 Bibliography

Identification Guide to European Non-Passerines. By K Baker (BTO Guide 24)
Seabirds - an identification guide. By Peter Harrison. Croom Helm, Kent. 1983
The Seabirds of Britain and Ireland. By S.Cramp, WRP Bourne & D Saunders. Country Book Society. 1974.
The Status of Seabirds in Britain and Ireland. By C. Lloyd, M. Tasker, & K Partridge. T & AD Poyser, London. 1991.
The Atlas of Wintering Birds in Britain and Ireland. By P. Lack. T & AD Poyser, London.1986.
The Atlas of Breeding Birds in Britain and Ireland. JTR Sharrock. T & AD Poyser, London. 1976.

Brandl, R. & Nelson, I. 1988. Feeding frequency of black-headed gull chicks. Bird Study. **35**, 137-141.

10.4 References

---

[i] From: Nice, M,M. 1962. Development of behaviour in precocial birds. Trans. Linn. Soc. N.Y. 8: 1-211.
[ii] Access the current list of British birds and their current names at the British Ornithological Union's website; http://www.bou.org.uk/recbrist1.html.
[iii] Grant P.J. 1982. Gulls - a guide to identification. T & A D Poyser, Staffordshire, England.
[iv] O'Connor RJ. 1984. The Growth and Development of Birds. John Wiley & Sons, England.
[v] Redfern, CPF. & Clark, JA. 2001. Ringers' Manual. BTO, Thetford.

© RSPCA Gulls 2013.docx
